# Cold-induced metabolic depression in cunner (*Tautogolabrus adspersus*): A multifaceted cellular event

**DOI:** 10.1371/journal.pone.0271086

**Published:** 2022-08-02

**Authors:** Lucie Gerber, Courtney E. MacSween, James F. Staples, A. Kurt Gamperl

**Affiliations:** 1 Department of Ocean Sciences, Memorial University of Newfoundland and Labrador, St. John’s, NL, Canada; 2 Department of Biology, University of Western Ontario, London, ON, Canada; National Cheng Kung University, TAIWAN

## Abstract

Metabolic depression and dormancy (i.e., stopping/greatly reducing activity and feeding) are strategies used by many animals to survive winter conditions characterized by food shortages and cold temperatures. However, controversy exists on whether the reduced metabolism of some fishes at cold temperatures is due to dormancy alone, or also involves active metabolic depression. Thus, we acclimated winter-dormant cunner [*Tautogolabrus adspersus*, a north temperate wrasse which in Newfoundland is at the northern limit of its distribution] and winter-active Atlantic salmon (*Salmo salar*) to winter (0°C; 8h light: 16h dark) and summer (10°C; 16h light: 8 h dark) conditions, and measured the thermal sensitivity of ATP-producing and O_2_-consuming processes in isolated liver mitochondria and hepatocytes when exposed *in vitro* to temperatures from 20 to 0°C and 10 to 0°C, respectively. We found that: 1) liver mitochondrial State 3 respiration and hepatocyte O_2_ consumption in cunner were only ~ one-third and two-thirds of that measured in salmon, respectively, at all measurement temperatures; 2) cunner mitochondria also have proton conductance and leak respiration (State 4) values that are only approximately one-third of those in salmon; 3) the mitochondria of cunner show a dramatic reduction in respiratory control ratio (from ~ 8 to 3), and a much greater drop in State 3 respiration, between 10 and 5°C (Q_10_ values in 10- and 0°C-acclimated fish of 14.5 and 141.2, respectively), as compared with salmon (3.9 and 9.6, respectively); and 4) lowering temperature from 5 to 0°C resulted in ~ 40 and 30% reductions in hepatocyte O_2_ consumption due to non-mitochondrial respiration and Na^+^-K^+^-ATPase activity, respectively, in cunner, but not in salmon. Collectively, these results highlight the intrinsic capacity for metabolic depression in hepatocytes and mitochondria of cunner, and clearly suggest that several cellular processes play a role in the reduced metabolic rates exhibited by some fishes at cold temperatures.

## Introduction

Metabolic depression involves the active reduction of energy-demanding processes of an organism, or in specific tissues, in the face of environmental stressors [1]. The extent of this regulated metabolic depression (which can occur when animals engage in torpor, hibernation, diapause or brumation), and the mechanisms that control/mediate it, have been extensively studied in mammals, frogs and turtles [1, 2], but are less understood in fishes in relation to cold stress [3, 4]. When exposed to cold temperatures, fish often become behaviorally inactive–a state termed dormancy [3, 5]. This is an effective way for many species to conserve energy stores and survive harsh environmental conditions [6]. However, there is evidence that some species of fish go even further, and also employ metabolic depression as a strategy to reduce metabolic costs in winter [4, 7]. Nonetheless, controversy exists as to whether fish that enter a dormant state during harsh winter conditions are simply inactive, or also employ active metabolic depression [8].

The evidence for cold-induced metabolic depression (hypometabolism) is largely based on the calculation of Q_10_ values for metabolism and other physiological functions; this metric is the fold change in the rate of a particular process over a 10°C change in temperature. When changes in a biological rate are caused by passive thermal effects, Q_10_ values generally range from 2–3, whereas Q_10_ values greater than 3.5 have been used to imply active metabolic regulation [4, 9]. For example, when water temperatures are lowered from 10 to ≤ 5°C, the smallmouth bass (*Micropterus dolomieu*) and brown bullhead (*Ictalurus nebulosus*) have Q_10_ values around ~ 2.5 for O_2_ consumption (MO_2_), whereas the eel (*Anguilla rostrata*) and the goldsinny wrasse (*Ctenolabrus rupestris*, L.) have Q_10_ values of 4.1 and 16, respectively [3, 10–12]. The latter Q_10_ values strongly suggest that metabolic depression is being utilized, in addition to inactivity, at least by some fish species to survive prolonged exposure to cold temperatures.

Research also suggests that the cunner (*Tautogolabrus adspersus*) uses active metabolic rate depression under these conditions. This species is a member of the mainly tropical family Labridae (the wrasses) that is distributed along the western Atlantic from Chesapeake Bay (USA) to Newfoundland (Canada, the northern limit of its distribution), where it enters a state of dormancy in the wild when seawater temperatures reach ~ 5°C [13]. A number of authors have reported Q_10_ values between 5 and 15 for MO_2_ (depending on sub-population and age / size) when cunner are exposed to acute or seasonal changes in temperature [14–18]. Further, Lewis and Driedzic [19] reported decreases in protein synthesis rates between 55 and 66% (depending on tissue) when temperature was lowered from 8 to 4°C (a Q_10_ of ~ 7.5). However, a recent study challenged whether this species engages in cold-induced metabolic depression. Speers-Roesch et al. [8] suggested that winter dormancy in cunner is not attributable to active metabolic depression, but simply inactivity due to the cold and darkness typical of their environmental niche during the winter. Hence, despite evidence for active metabolic depression at the whole animal and tissue levels in cunner, and that several authors have shown that metabolic depression (not activity) is responsible for the lower metabolic rates of numerous fish species when exposed to other environmental stressors [20, 21], uncertainty remains on whether winter dormancy in fishes is associated with an active depression of metabolic activities.

To achieve a state of metabolic depression, some ATP-producing and–utilizing processes must be turned down, and a new balance between the rates of ATP production and O_2_ consumption must be established. Several processes with high cellular ATP demand (i.e., which result in substantial cellular O_2_ consumption), are therefore, ideal cellular targets for energy savings during metabolic depression [4]; these include protein synthesis (which comprises between 20–80% of cellular respiration) and ion pumping (which makes up to 40% of cellular energy demands; e.g., Na^+^-K^+^-ATPase activity). These costly cellular processes are known to be reduced significantly during harsh environmental conditions in various taxa, and to contribute to metabolic depression (see reviews and references therein [1, 7, 9, 22–24]). Finally, there are a number of reactions independent of mitochondrial ATP synthesis that consume O_2_, and would be predicted to be decreased greatly during metabolic depression. For instance, values of non-mitochondrial respiration as high as 70% of cellular respiration have been reported in trout (*Oncorhynchus mykiss*; [25].

In the present study, we measured the effect of thermal acclimation (0 and 10°C) and acute exposure to a range of temperatures (0–20°C) *in vitro* on many of the cellular processes described above in cunner and Atlantic salmon (*Salmo salar*, a species that remains active in winter). This allowed us to examine whether the former species has the potential to utilize metabolic depression *in vivo* when exposed to winter conditions (i.e., at low temperatures and limited day length). This research focused on isolated liver mitochondria and hepatocytes as this organ accounts for approximately 35–50% of an organism’s standard metabolic rate, and was identified in various taxa as a target for metabolic depression [26–29]. Specifically, we assessed: 1) State 3 and 4 respiration (i.e., oxygen consumption due to oxidative phosphorylation and proton leak, respectively) and measures of efficiency [i.e., the respiratory control ratio (RCR) and P:O ratio] in isolated mitochondria at 1, 5, 10 and 20°C; 2) the kinetics of mitochondrial proton leak at 5 and 20°C; and 3) total, non-mitochondrial and Na^+^-K^+^-ATPase-dependent O_2_ consumption by hepatocytes at 0, 5 and 10°C.

## Materials and methods

### Animals

Adult cunner were collected by the Ocean Sciences Centre’s field services unit in Conception Bay (NL, Canada; 47.5° N, 52.8° W), whereas juvenile Atlantic salmon were obtained from a commercial grower (Cooke Aquaculture, Daniels Harbour, NL, Canada) and were > 200g at the time of experimentation (Table 1). Prior to experimentation, the fish were kept in separate 1 m^3^ tanks and held for at least ten weeks under either ‘summer’ (10 + 0.5°C and 16h light: 8h dark) or ‘winter’ (0 ± 0.2°C and 8h light: 16h dark) conditions to allow for complete temperature / seasonal acclimation. During this period, the cunner were fed chopped herring twice weekly, while the salmon were fed a commercially available pelleted diet (Europa; Skretting Inc.) daily.

**Table 1:** Morphometric parameters measured in 0 and 10°C acclimated cunner and salmon. Letters indicate a significant difference between groups, whereas a plus sign (^+^) indicates a significant acclimation effect (P < 0.05) as determined by 2-way ANOVA. Values are means ± s.e.m., N = 7–9.

	Cunner		Salmon	
	0°C Acclimated	10°C Acclimated	0°C Acclimated	10°C Acclimated
**Length (cm)**	24.2 ± 0.8^a^	24.3 ± 0.8^a^	47.6 ± 1.1^b^	46.5 ± 1.1^b^
**Body Mass (g)**	195.2 ± 19.9^ac^	196.1 ± 22.1^a^	293.9 ± 17.3^b^	257.1 ± 22.7^c^
**Liver Mass (g)**	3.1 ± 0.3^a^	4.8 ± 0.5^b^	5.4 ± 0.5^b^	4.39 ± 0.5^b^
**Hepatic Somatic Index (HSI)**	1.6 ± 0.1^a+^	2.5 ± 0.2^a^	1.9 ± 0.2^a^	1.7 ± 0.2^a^

All experimental studies were conducted in accordance with the guidelines published by the Canadian Council on Animal Care, and approved by the Animal Care Committee at Memorial University of Newfoundland (protocol ^#^07-10-KG).

### Morphometric variables

Fish were anaesthetized using tricaine methanesulfonate (TMS, 0.2 g L^-1^; Syndel Laboratories, Vancouver, B.C.), and then euthanized with a blow to the head. For each fish, body length, mass and liver mass were measured. The hepatosomatic index (HSI; i.e., the ratio of liver mass to body mass) was used as an indicator of the energy state of fish acclimated to 0 *vs*. 10°C.

### Mitochondrial isolation

Mitochondrial isolation followed a slightly modified protocol from that outlined in [30, 31]. A small portion of liver was frozen immediately in liquid N_2_, and subsequently stored at -80°C until later measurement of citrate synthase (CS) activity. The rest of the liver was used for mitochondrial isolation. The osmolality of the respiration medium for the 10°C acclimated cunner, and 10°C and 0°C acclimated salmon, was 335 mOsm and corresponded to that determined in preliminary measurements on plasma [Wescor vapor osmometer (Model 5520)]. However, 0°C acclimated cunner had a plasma osmolality of 380 mOsm and the respiration medium was modified to reflect this difference. This ensured viable mitochondrial preparations for all groups. For mitochondrial isolation, the liver was rinsed with ice-cold isolation buffer (in mmol L^-1^: 230 Mannitol, 75 Sucrose, 1 EGTA, 20 HEPES, with 2% fatty acid free albumin; expect for 0°C acclimated cunner where 275 mmol L^-1^ mannitol was used) and minced using a razor blade. The minced liver was then homogenized by passage through a loose fitting Teflon^®^ pestle (Wheaton, Millville USA; clearance approx. 2 mm), three times at 100 RPM. The resulting homogenate was subsequently filtered through one layer of cheesecloth and centrifuged at 800 *xg* for 10 min at 1°C. Lipids were then aspirated off, and the remaining supernatant filtered through four layers of cheesecloth and centrifuged again at 800 *xg* for 10 min at 1°C. This procedure was repeated twice. The obtained supernatant was then centrifuged at 5750 *xg* for 10 min at 1°C. The pellet containing mitochondria was washed by re-suspension in excess isolation buffer and centrifuged again at 5750 *xg* for 10 min at 1°C. The final pellet was re-suspended in 1 mL of respiration buffer (in mmol L^-1^: 160 KCl, 30 HEPES, 10 KH_2_PO_4,_ 2 EDTA, 1 MgCl_2,_ with 1% fatty acid free albumin; expect for 0°C acclimated cunner where 180 mmol L^-1^ KCl was used). A small volume (50 μL) of the mitochondrial re-suspension was snap-frozen in liquid N_2_, and subsequently stored at -80°C for determination of protein content and CS activity. The rest of the mitochondrial suspension was kept on ice for less than 2 hours prior to the measurement of mitochondrial respiration and proton leak kinetics.

### Mitochondrial respiration

Mitochondrial respiration was measured using custom made, water-jacketed, glass respiration chambers (2 mL in volume) fitted with a Delrin^®^ plunger and containing a mini glass-coated stir bar. After adding 1.9 mL of assay buffer and 100 μL of mitochondrial re-suspension (final concentration ~ 0.3 mg protein mL^-1^) to the chamber, O_2_ levels were measured using a pre-calibrated (with 100% air saturated water and 1 g L^-1^ sodium sulfite) PreSens (Regensburg, Germany) fiber-optic O_2_ sensor spot (Cat. ^#^ SP-PSt3-NAU-DS-YOP) glued onto the inside of the respiration chamber, and a Fibox 3 mini-sensor O_2_ meter (PreSens) with a fiber-optic light guide. Data were continuously recorded using PreSens software (Version PST3 v532.exe) at 1 measurement every 10 sec. Mitochondrial respiration was assessed at a range of temperatures (1, 5, 10, and 20°C) experienced by fish in their natural environment. Before measurement of mitochondrial respiration, the mitochondria were allowed to equilibrate (for 10 to 20 min) in the chamber at each experimental temperature. The order of the temperature assays was randomized to ensure that post-isolation time did not affect the results.

Each assay was initiated by addition of the substrates malate (1.5 mmol L^-1^) and glutamate (7.5 mmol L^-1^) to the chamber (State 2 respiration). After 10 min, State 3 respiration was initiated by addition of ADP (0.2 mmol L^-1^) to measure O_2_ consumption during oxidative phosphorylation, and State 4 respiration was measured once all the added ADP had been phosphorylated.

### Mitochondrial proton leak kinetics

Measurements of mitochondrial membrane potential (Δψ_mt_) were performed in the same respiration chamber, but in separate experiments, and followed the methodologies outlined in [26, 30]. Briefly, Δψ_mt_ was estimated using tetraphenylphosphonium (TPP^+^), a lipophilic cation whose uptake by mitochondria depends on membrane potential [32]. A TPP^+^ selective electrode (World Precision Instruments, Sarasota FL) and a reference electrode were inserted through the Delrin^®^ plunger and connected to an Accumet Excel XL15 Series pH meter (Fisher Scientific, Bartlesville, OK) to measure TPP^+^ concentration ([TPP^+^]) in the respiration chamber. Experiments were carried out at two of the experimental temperatures used for mitochondrial respiration (i.e., 5 and 20°C; measurements at 0°C were not possible due to a poor signal to noise ratio). Prior to the addition of mitochondria, the TPP^+^ selective electrode was calibrated by incremental additions of TPP^+^ (1 μmol L^-1^) at each experimental temperature. Then, mitochondrial suspension (150 μL; ~ 0.45 mg protein mL^-1^) was added to the chamber containing 1.85 mL of respiration buffer, and rotenone (a Complex I inhibitor, 0.1 mmol L^-1^) and oligomycin (a Complex V inhibitor, 1.5 μmol l^-1^) were subsequently added to the chamber to inhibit O_2_ consumption related to ATP synthesis. Then, succinate [Complex II (CII) substrate, 6 mmol L^-1^] was added to fuel mitochondrial leak respiration *via* CII. Mitochondrial O_2_ consumption and mitochondrial membrane potential (Δψ_mt_) were then recorded simultaneously, and continuously, during the stepwise inhibition of substrate (succinate) oxidation by titration with malonate, a CII competitive inhibitor (5 x 0.33 mmol L^-1^, final concentration 1.65 mmol L^-1^)_._ Finally, CCCP (0.1 μmol L^-1^) was added to uncouple the mitochondria and allow for correction of TPP^+^ electrode drift. To determine proton leak kinetics, succinate-fueled mitochondrial O_2_ consumption was plotted against mitochondrial membrane potential (Δψ_mt_). In this analysis, a higher level of O_2_ consumption at a particular Δψ_mt_ is indicative of increased proton leak / conductance [33]. Δψ_mt_ was calculated using a modified Nernst equation [26, 30] and assuming the mitochondrial matrix volume was 0.001 mL mg^-1^ protein.

### Hepatocyte isolation

Hepatocyte isolation followed the general protocol outlined in [34], with some modifications. Briefly, fish were anesthetized using TMS and killed with a blow to the head. The liver was then cannulated through the hepatic vein, and perfused in a retrograde direction starting with a ~ 15°C HEPES buffered saline solution (in mmol L^-1^: 176 NaCl, 5.4 KCl, 0.81 MgSO_4_, 0.44 KH_2_PO_4_, 0.35 Na_2_HPO_4_, 5 NaHCO_3_, 10 HEPES) with EGTA (1 mmol L^-1^), and then by HEPES buffered saline containing Type IV collagenase (0.3 mg mL^-1^). Once the liver was completely digested, it was filtered through fine nylon mesh (100 μm) using up to 50 mL of washing solution (HEPES buffered saline solution with 2% fatty acid free albumin and 1.5 mmol L^-1^ CaCl_2_). The obtained cell suspension was centrifuged at 60 *xg* for 4 min at 4°C. The supernatant was discarded, and the cells re-suspended in 5 ml of washing solution. This process was repeated twice until the supernatant was free of most cellular debris. Then, the cells were suspended in 2 mL of washing solution and centrifuged one final time at 60 *xg* for 4 min at 4°C. The supernatant was again discarded, and the cells re-suspended in assay solution (i.e., washing solution + 5 mmol L^-1^ glucose) at a cell concentration of approximately 20–40 x 10^6^ cells mL^-1^. Cell numbers and viability were determined using a Neubauer hemocytometer and Trypan blue exclusion, respectively. Finally, the cells were placed in an incubator at the fish’s acclimation temperature for 2 hours to ‘rest’ before determination of total, non-mitochondrial and Na^+^-K^+^-ATPase-dependent O_2_ consumption by the hepatocytes. O_2_ consumption (nmol O min^-1^ 10^6^ cells) was measured at 0, 5 and 10°C using the calculations described above, except that protein concentration was replaced by cell concentration (10^6^).

### Total, non-mitochondrial and Na^+^-K^+^-ATPase-dependent O_2_ consumption by hepatocytes

Hepatocyte O_2_ consumption was determined in the respiration chambers as described above. Hepatocyte suspensions (250 μL, ranging from ~ 20–40 x 10^6^ cells) were added to the chamber which already contained 1.75 mL of assay solution and O_2_ consumption was determined after a 15–20 min stabilization period. Then stepwise additions (up to 3) of myxothiazol (0.1 mg mL^-1^), an inhibitor of mitochondrial cytochrome c reductase, were performed to ensure the complete inhibition of mitochondrial respiration and prevent any O_2_ consumption associated with mitochondrial ATP synthesis. Complete inhibition of mitochondrial respiration was determined as the point where further additions of myxothiazol failed to change hepatocyte O_2_ consumption. Non-mitochondrial O_2_ consumption (i.e., O_2_ consumption related to other cell processes) was then measured as the difference in O_2_ consumption before and after the addition of myxothiazol.

Hepatocyte O_2_ consumption dependent on Na^+^-K^+^-ATPase activity was measured on separate hepatocyte suspensions before and after the addition of ouabain (1 mmol L^-1^), a specific inhibitor of Na^+^-K^+—^ATPase activity.

### Citrate synthase activity

To estimate mitochondrial density and aerobic capacity, CS activity was measured in both isolated mitochondria and whole liver tissue. Frozen liver tissue was thawed in 9 volumes of ice-cold grinding buffer (in mmol L^-1^: 25 HEPES, 2 EDTA and 0.1% Triton X-100, pH 7.0), minced thoroughly with scissors, and then homogenized with a Tissue Tearor (Biospec Inc., Bartlesville, OK) for 3 x 10 sec, with 30 sec ‘rest’ on ice in between each homogenization. The homogenate was then centrifuged at 4°C for 5 min at 2000 *xg*, and the supernatant used to assay CS activity. Isolated mitochondria were thawed and diluted in 9 volumes of the same ice-cold grinding buffer and vortexed for 10 sec prior to the determination of CS activity. CS activity was measured at room temperature (22°C) using a SpectraMax M2^e^ (Molecular Devices, Sunnyvale, CA) microplate reader. To perform this assay, 231 μL of buffer (50 mmol L^-1^ Tris, pH 8.0 at room temperature), 30 μL of DTNB (0.15 mmol l^-1^), 30 μL of acetyl CoA (0.15 mmol L^-1^), and 6 μL of mitochondrial re-suspension (or 3 μL of liver supernatant) were added to individual wells of a 96-well plate. Then 3 μL of Tris buffer was added to the ‘control’ wells whereas all other wells received 3 μL of oxaloacetate (a metabolic intermediate of the citric acid cycle, 0.33 mmol L^-1^) to assess CS activity of the mitochondria and liver. The reaction was followed at 412 nm for 5 min (ε = 13.6 mmol L^-1^ cm^-1^), and activity was expressed as mU mg protein^-1^ or mU g wet liver^-1^.

### Chemicals

Chemicals used in these experiments were purchased from Sigma Aldrich (St. Louis, MO), Fisher Scientific (Pittsburgh, PA) and BDH Chemicals (Poole, England).

### Statistical analyses

For all measurements, the sample size (n) was 6–9. Morphometric variables [body length and mass, liver mass, and hepatosomatic index (HSI)], as well as liver and mitochondrial CS activity, were analyzed using 2-way ANOVAs (main effects: species and acclimation temperature). The mitochondrial respiration and hepatocyte O_2_ consumption data were analyzed using 2 (species) x 2 (acclimation temperature) x 4 or 3 (assay temperature) factorial analyses. Further, when there were significant interactions between assay temperature and species, 2–way ANOVAs were performed at each assay temperature to examine acclimation and species effects. These statistical tests for the effects of acclimation were followed by Neuman-Keuls tests. All data are reported as means ± standard errors of the mean (s.e.m.) and statistical analyses were performed using STATISTICA (StatSoft Inc., Tulsa, OK, USA) with P < 0.05 used as the level of statistical significance.

## Results

### Morphometric values

The length and mass of the salmon used in these experiments were greater than that for the cunner. Cunner acclimated at 0°C had smaller livers (3.1 g) than the other three groups (range ~ 4.4–5.4), but the HSI values were generally comparable (Table 1).

### Mitochondrial respiration

**Fig 1:**
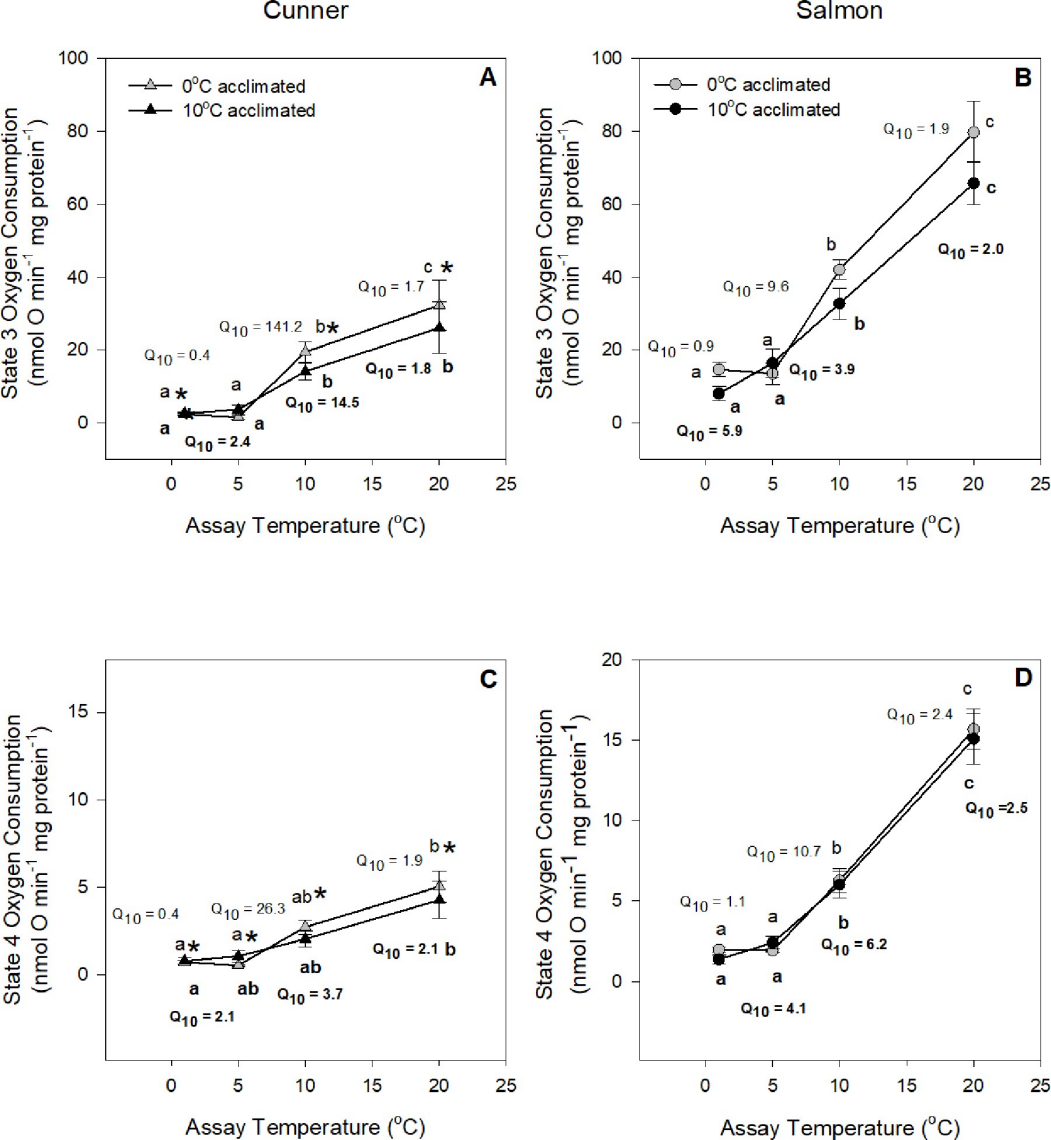
State 3 (A, B) and State 4 (C, D) respiration of isolated liver mitochondria from 0°C and 10°C acclimated cunner (A, C) and salmon (B, D), measured at 0°C, 5°C, 10°C and 20°C. Letters indicate significant differences within a species amongst assay temperatures, whereas an asterisk (*) indicates a significant difference between species at a particular assay temperature (P < 0.05); as determined by a 3-way repeated measures ANOVA, followed by 2-way ANOVAs and Newman-Keuls post-hoc tests. There were no significant acclimation effects. Q_10_ values calculated over the ranges 0–5°C, 5–10°C and 10–20°C are reported. Q_10_ values and letters in **bold** refer to the 10°C acclimated groups. Values are means ± s.e.m., N = 6–9.

Acclimation temperature (0 *vs*. 10°C) had no overall effect on either State 3 or State 4 mitochondrial respiration at any particular assay temperature. However, State 3 and 4 respiration were consistently lower in cunner as compared with salmon. Further, acclimation temperature and species had a considerable, and varied, influence as assay temperature was lowered from 20 to 10°C, from 10 to 5°C, and finally from 5 to 0°C (Fig 1).

At 20°C, State 3 and 4 O_2_ consumption for cunner mitochondria were approximately one-third of those measured for salmon (24 and 4 nmol O min^-1^ mg protein^-1^ *vs*. 71 and 15 nmol O min^-1^ mg protein^-1^, respectively), and these variables declined at similar rates in both species as assay temperature was lowered from 20 to 10°C (Q_10_ values ranging from 1.7 to 2.5). In contrast, when temperature was reduced from 10 to 0°C, the magnitude of the decrease in these two variables was different, and species- and acclimation temperature-dependent. Overall, a number of general patterns could be described: (1) The relative decrease in respiration rates (represented by Q_10_ values) was greatest between 10 and 5°C, and much greater for cunner over this temperature range. (2) Although changes in State 3 and 4 respiration for salmon were similar as temperature was lowered from 10 to 5°C (Q_10_ values of 4–9), State 3 respiration fell much more than State 4 in cunner (e.g., Q_10_ values were 141.2 *vs*. 26.3, respectively, for 0°C acclimated cunner). This resulted in values of State 3 and State 4 respiration for cunner that were approx. one-tenth and one-third, respectively, of those measured in salmon mitochondria at 5°C. (3) For cunner, State 3 and 4 respiration fell more in 0°C acclimated fish between 10 and 5°C (Q_10_ values > 140 for 0°C acclimated cunner *vs*. < 15 for 10°C acclimated cunner), but less between 5 and 0°C (Q_10_ values < 0.5 for 0°C acclimated cunner *vs*. > 2 for 10°C acclimated cunner). The net result was that mitochondria from 0°C cunner were better able to maintain their metabolic rate when assay temperature was reduced from 5 to 0°C. (4) Although 0°C acclimated salmon were also able to maintain mitochondrial respiration as water temperature was lowered from 5 to 0°C, a different response was observed for 10°C acclimated salmon. Mitochondria from these fish had Q_10_ values for O_2_ consumption of ~ 4–6 from 5 to 0°C; values approx. twice those measured in cunner. This latter result indicates that 10°C acclimated cunner are better than 10°C acclimated salmon in maintaining mitochondrial respiration when challenged with 0°C seawater.

**Fig 2:**
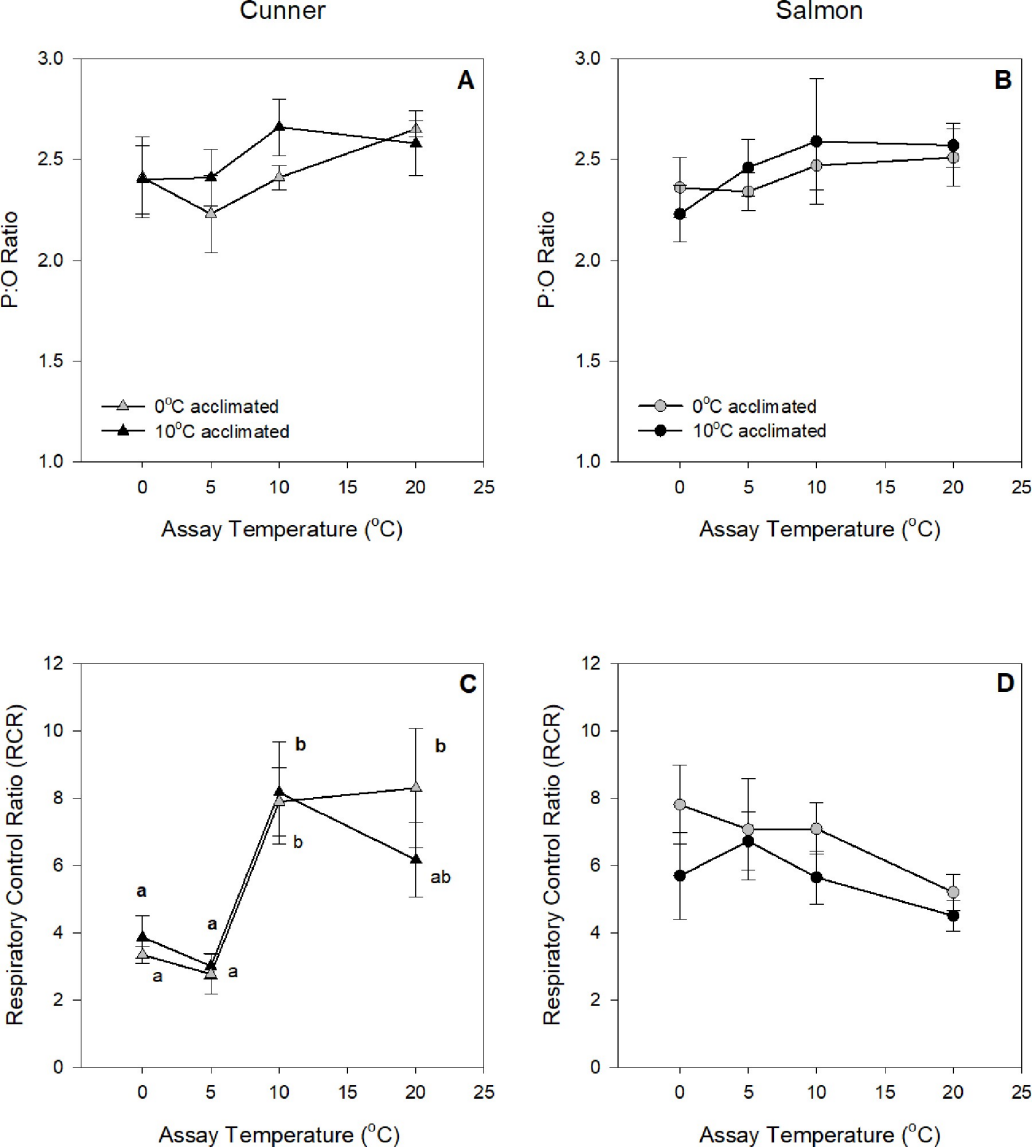
P:O ratio (A, B) and respiratory control ratio (RCR; C, D) values for isolated liver mitochondria from 0 and 10°C acclimated cunner (A, C) and salmon (B, D), measured at 0°C, 5°C, 10°C and 20°C. Letters indicate significant differences within a species amongst assay temperatures, whereas an asterisk (*) indicates a significant difference between species at a particular assay temperature (P < 0.05). There were no significant acclimation effects or significant differences between species at a particular assay temperature; as determined by a 3-way repeated measures ANOVA, followed by 2-way ANOVAs and Newman-Keuls post-hoc tests. Values without a letter in common are significantly different within a species between assay temperatures. Q_10_ values and letters in **bold** refer to the 10°C acclimated groups. Values are means ± s.e.m., N = 7–9.

The P:O ratio calculated during State 3 mitochondrial respiration ([ADP_injected_ in μmol]/[O_2 consumed_ in μ-atoms]) was used to assess the efficiency of ATP synthesis. The P:O ratio (Fig 2A and 2B) was not altered by thermal acclimation or assay temperature, and was not different between the two species (range ~2.2–2.5).

The respiratory control ratio (RCR; State 3/State 4), which indicates how well mitochondrial substrate oxidation is coupled to ADP phosphorylation, was not altered by thermal acclimation in either species (Fig 2C and 2D). In cunner, the RCR was similar at 20°C and 10°C, but was reduced by ~50% (from ~8 to ~4) between 5°C and 0°C (Fig 2C). RCR for the salmon mitochondria was not altered by assay temperature, and ranged from 5 to 7.5 (Fig 2D); these values were similar to that of the cunner at 20°C and 10°C.

### Proton leak kinetics

**Fig 3:**
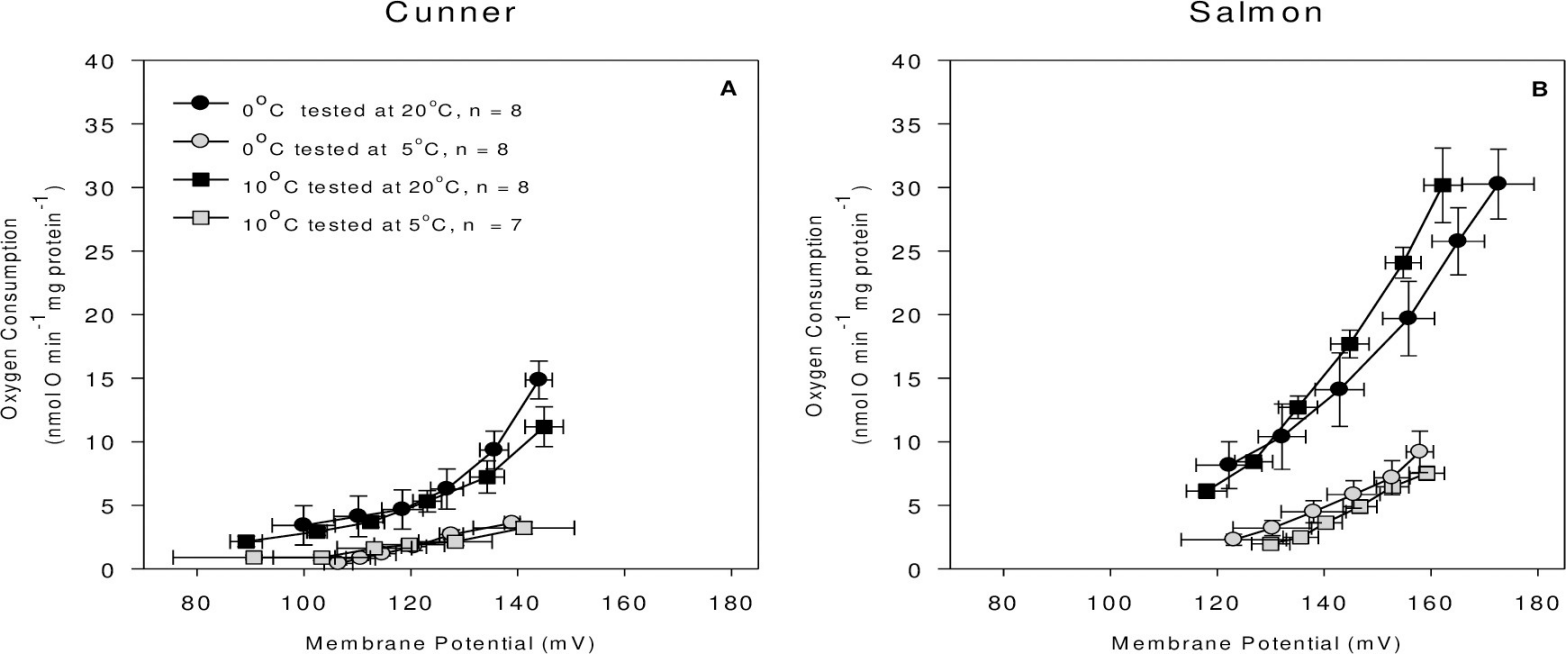
Kinetics of proton leak. Parallel measurements of O_2_ consumption (i.e., succinate oxidation) and membrane potential of isolated liver mitochondria from 0 and 10°C acclimated cunner (A) and salmon (B), measured at 5°C and 20°C. Maximal proton leak rates are indicated by the top right-most points. There were no significant acclimation effects. Values are means ± s.e.m., N = 7–8.

The relationship between succinate-fueled mitochondrial O_2_ consumption (leak respiration) and mitochondrial membrane potential (Δψ_mt_) was used to assess the kinetics of proton leak at 20°C and 5°C in liver mitochondria from 0°C and 10°C acclimated cunner and Atlantic salmon (Fig 3). Acclimation temperature only had a minor effect on proton leak kinetics, and only at an assay temperature of 20°C. Acclimation to 0°C resulted in an increase in O_2_ consumption in cunner at the same Δψ_mt_, whereas salmon were able to achieve a higher Δψ_mt_ at the same O_2_ consumption. Proton leak kinetics were also altered by assay temperature in both species, with ‘leakier’ mitochondria at 20°C as compared to 5°C (i.e., higher Δψ_mt_ and O_2_ consumption). At 20°C, the State 4 respiration of cunner mitochondria was approx. one-third of that measured in salmon (indicating decreased respiratory chain activity), and there was a leftward shift in the proton leak curve reflecting a decrease in proton conductance. For example, at a Δψ_mt_ of 130 mV, State 4 O_2_ consumption at 20°C was approx. 40% lower in the cunner (i.e., 6 *vs*. 11 nmol O min^-1^ mg protein^-1^). Differences were also apparent at an assay temperature of 5°C, despite the 3.5-fold difference in State 4 respiration as compared to when these variables were measured at 20°C. However, at 5°C, the curve for cunner appears to simply be an extension of the data obtained for salmon at this temperature. These data suggest that proton leak kinetics differed between the two species at this assay temperature due to reduced respiratory chain activity alone (i.e., proton conductance was not different).

### Hepatocyte, non-mitochondrial and Na^+^-K^+^-ATPase dependent O_2_ consumption

**Fig 4:**
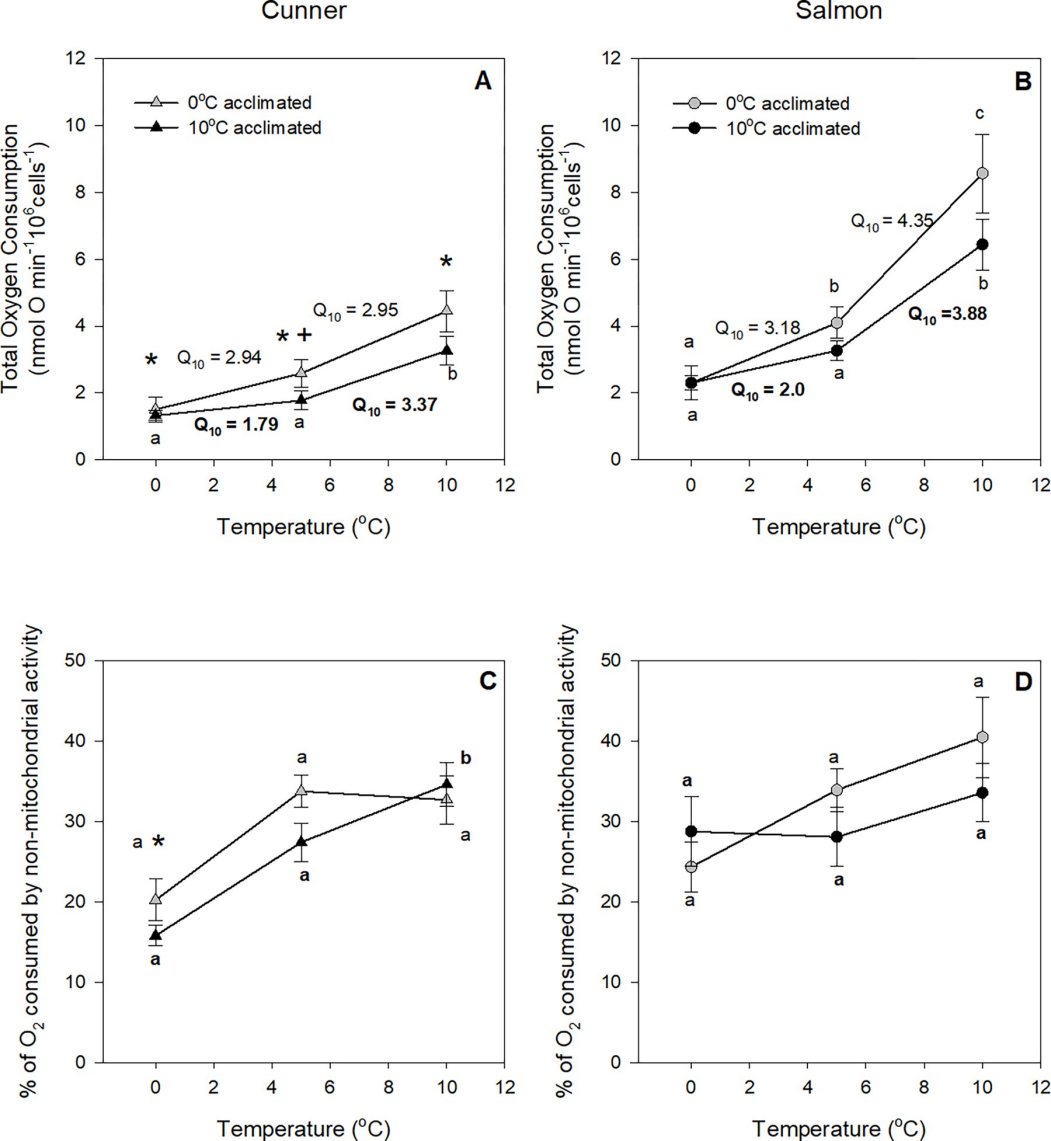
Total O2 consumption of isolated hepatocytes (A, B) and the percentage of O_2_ consumed by non-mitochondrial activities (C, D) and by Na^+^-K^+^-ATPase activity (E, F) of isolated hepatocytes from 0 and 10°C acclimated cunner (A, C) and salmon (B, D), measured at 0°C, 5°C and 10°C. Letters indicate significant differences within a species between assay temperatures whereas an asterisk (*) and a plus sign (+) indicate a significant difference between species at a particular assay temperature at P < 0.05 and 0.05 < P < 0.1; as determined by a 3-way repeated measures ANOVA, followed by 2-way ANOVAs and Newman-Keuls post-hoc tests. There were no significant acclimation effects. Q_10_ values and letters in **bold** refer to 10°C acclimated groups. Values are means ± s.e.m., N = 7–8.

In agreement with the data collected on isolated mitochondria, acclimation temperature had no effects on hepatocyte O_2_ consumption measured at any assay temperature, and the difference in O_2_ consumption between salmon and cunner hepatocytes at 10°C was approx. 2-fold (i.e., compare Figs 1A, 1B, 4A and 4B). However, this is where the similarity between isolated mitochondria and hepatocytes ended. Hepatocyte O_2_ consumption did not drop sharply as assay temperature was lowered from 10 to 5°C, and was not species-dependent (Q_10_ values ranging from 2.2 to 4.5). Although the decrease in O_2_ consumption between 5 and 0°C was generally less than observed between 10 and 5°C, the difference in Q_10_ values was minimal (mean Q_10_ values 3.4 and 2.8, respectively). Finally, acclimation temperature did not influence the pattern of change in hepatocyte O_2_ consumption as assay temperature was lowered from 10 to 5°C, and then 5 to 0°C. The net result was that the difference in hepatocyte O_2_ consumption between salmon and cunner at 0°C was similar to that measured at 10°C.

Acclimation temperature had no effect on hepatocyte non-mitochondrial or Na^+^-K^+^-ATPase -dependent O_2_ consumption. Non-mitochondrial O_2_ consumption was approx. 30–40% of total cellular respiration in salmon at all temperatures, and in cunner at 10 and 5°C. However, this variable fell to 20% in cunner hepatocytes at 0°C, and this difference was significant as compared to salmon hepatocytes measured at 0°C and cunner hepatocytes assayed at 10°C (Fig 4C and 4D). The percentage of O_2_ consumption dependent of Na^+^-K^+^-ATPase activity was approx. 20% in most species-assay temperature combinations, except for cunner hepatocytes at 0°C where it was approx. 15% (Fig 4E and 4F) and significantly lower than that of salmon at 0°C.

### Citrate synthase activity

Liver CS activity was comparable between the two species. In contrast, the liver mitochondrial CS activity of cunner was approx. one-half that of salmon. There was no effect of acclimation temperature on liver or mitochondrial CS activity in either species (Table 2).

**Table 2:** CS activity in isolated mitochondria and liver from 0 and 10°C acclimated cunner and salmon. Letters indicate a significant difference between groups; as determined by a 2-way ANOVA followed by Newman-Keuls post-hoc tests. There were no significant acclimation effects. Values are means ± s.e.m., N = 6–9.

	Cunner		Salmon	
	0°C Acclimated	10°C Acclimated	0°C Acclimated	10°C Acclimated
Liver CS activity (U g tissue^-1^)	3.4 ± 0.2^a^	3.2 ± 0.4^a^	3.5 ± 0.4^a^	4.6 ± 0.3^a^
Mitochondrial CS activity (mU mg protein^-1^)	107.5 ± 11.8^a^	121.5 ± 11.8^a^	206.7 ± 17.9^b^	226.2 ± 33.6^b^

## Discussion

Whether, and to what extent, active metabolic suppression is associated with the ‘dormant’ state in cunner at cold temperatures (i.e., in winter) is controversial. A recent study suggests that inactivity alone is responsible for this dormant state, and does not involve / require active metabolic depression [8] as concluded by earlier behavioral and physiological studies [14–19, 35]. Here, we examined the thermal sensitivity of ATP-consuming and -producing processes in an essential metabolic tissue, the liver, of two marine fishes that employ different strategies (activity *vs*. dormancy) to survive harsh winter conditions. The Atlantic salmon and cunner are both year-round residents in Newfoundland coastal waters, and have similar optimal temperatures (between 8–15°C) [13, 36], but respond differently to the annual temperature variations found in this habitat. During the cold winter months where sea temperatures remain below 5°C, Atlantic salmon remain active around Newfoundland whereas cunner enter into a dormant state and are inactive [16, 37]. The Atlantic salmon, is therefore, an excellent cold-active model to compare with the cold-inactive cunner with regard to investigating whether metabolic depression is employed by the cunner to survive winter harsh condition, the magnitude of the metabolic depression, and what cellular processes might be modified to save energy at cold temperatures.

### Metabolism in winter dormant cunner vs. winter active Atlantic salmon

At all assay temperatures (20, 10, 5 and 1°C), cunner had much lower mitochondrial respiration rates (by ~50%) than those of salmon (Fig 1). The ability of cunner to downregulate their metabolism may seem surprising given that they already have a much lower MO_2_ [16, 37]. However, the total O_2_ consumed by isolated hepatocytes from cunner was also lower (by ~ 25–30%) than that of salmon at all four assay temperatures, and the liver mitochondrial respiration rates reported here for cunner and salmon fall in line with the previously reported liver mitochondrial respiration rates of winter-inactive (seabass, *Dicentrarchus labrax*; [38]) and -active (rainbow trout, *Oncorhynchus mykiss*; [39]) fish species, respectively. Interestingly, mitochondrial CS activity in cunner was also half that of salmon, whereas tissue (liver) CS activity was comparable (Table 2). This suggests that while cunner liver mitochondria have a lower CS activity as compared to salmon, tissue mitochondrial density is actually higher in the former species. On the other hand, while cunner non-mitochondrial and Na^+^-K^+^-ATPase-dependent O_2_ consumption rates were comparable to that of salmon at 5–20°C, they were ~ 30% lower in cunner at 0°C compared to salmon (Fig 4), and the cunner consistently had lower mitochondrial leak respiration rates (State 4; Fig 1). Indeed, this latter finding was confirmed by studying the kinetics of proton leak. When the curves were compared, the cunner had both lower O_2_ consumption rates and lower mitochondrial membrane potential, indicating that they expend less energy to maintain such potentials (i.e., their mitochondria are not as ‘leaky’; Fig 3). Collectively, these results illustrate that cunner have a lower requirement for energy when compared to the salmon, independent of temperature.

### Cold-induced metabolic depression

The thermal sensitivity of biological processes (Q_10_) is commonly used as evidence of metabolic depression. The physiochemical effects of temperature on biological processes are identified by Q_10_ values in the range of 2–3, although some authors use a Q_10_ of < 3.5 [9]. This simple temperature effect has been observed in the metabolic rate of temperate, tropical and polar teleost fishes within their normal thermal range [40–42]. While the effect of temperature on the routine metabolic rate of cunner is of this magnitude between 9 and 14°C (Q_10_ = 3.1; [16]) and 12 and 22°C (Q_10_ = 2.4; [17]), it is when temperatures fall to 5–7°C that we begin to see an active down-regulation of routine metabolic rate in this species, as indicated by Q_10_ values > 3.5 [16, 18]. This temperature onset of reduced metabolic rate corresponds with what was observed in this study. Indeed, the greatest relative reduction in liver mitochondrial respiration (Q_10_) was when temperature was reduced from 10 to 5°C (Fig 1). This provides additional, and convincing, evidence that the cunner utilizes metabolic depression to withstand low environmental temperatures. It was also between 10 and 5°C that the RCR values for cunner mitochondrial respiration fell by half, and this was due to a much greater drop in State 3 than State 4 respiration (Fig 2C). These data confirm a loss of mitochondrial oxidative capacity. In fact, the impressive Q_10_ values observed for cunner mitochondrial State 3 respiration when temperature was reduced from 10 to 5°C (e.g., 14 and 140 for 10 and 0°C acclimated cunner, respectively) indicate that there is tremendous capacity to suppress mitochondrial respiration in this species. Further: these temperatures correspond to the temperature range (~ 5–7°C) at which behavioral inactivity [13] and metabolic depression in cunner were initiated based on previous reports of down-regulation of whole organism metabolic rate [15, 16, 18] and protein synthesis [19, 35]; and the magnitude / degree of metabolic depression observed for cunner liver mitochondria in the present study has been reported for a diverse range of taxa that utilize metabolic depression during hibernation and torpor (see references in [41]). Interestingly, elevated Q_10_’s for mitochondrial respiration were also found for the 10°C-acclimted salmon, however, these tended to occur towards the lower end of this species thermal niche (i.e., between 5 and 0°C) and were less than measured in cunner [e.g., Q_10_ values for State 3 mitochondrial respiration were 14-fold higher in cunner than in salmon acclimated at 10°C when temperature was decreased from 10 to 5°C (Fig 1)]. It is noteworthy that as temperature dropped from 5 to 0°C, the effect of temperature became negligible in 0°C-acclimated cunner (Q_10_ value of 0.4). This confirms that the critical temperature with regards to reductions in metabolic rate is between 10 and 5°C for this species, and illustrates the fine-tuning of systems for cell and energy maintenance (i.e., ATP consumption and production).

Nonetheless, it was remarkable to see that the intrinsic reduction in O_2_ consumption at the mitochondrial / organelle level was not observed / conserved at the hepatocyte / cellular level. Indeed, the Q_10_ value of hepatocyte respiration as assay temperatures was reduced from 10 to 5, and then to 0°C, was fairly constant in both species (Q_10_ of ~ 3 at all tested temperatures in both species; Fig 4). Although, it must be recognized that 10°C acclimated cunner have a Q_10_ of ~ 4 for hepatocyte O_2_ consumption, and that this suggests that they have some capacity for metabolic depression, which was not observed for 0°C-acclimated cunner (Q_10_ of ~ 2). These results suggest that the cell can exercise control over individual components and processes, and ‘compensate’ for the direct effects of temperature on mitochondrial respiration. With regards to the cunner, the liver is involved in the production / synthesis of antifreeze proteins to ensure survival at near-freezing temperatures where potential contact with ice-laden seawater exists [43, 44]. Indeed, the liver seems to undergo a hyperactivation with regard to the synthesis of proteins when temperature falls from 4 to 0°C in cunner [19]. This hyperactivation of liver protein synthesis could explain the absence of clear active metabolic suppression in hepatocytes reported in the present study. Another possible explanation for the lack of evidence supporting active metabolic suppression, and much lower overall rates of O_2_ consumption by the hepatocytes, could be the fact that these *ex vivo* cells are missing some important cues (i.e., hormones) from the more integrated, *in vivo*, system. Combined, these data indicate that mitochondria likely have some inherent capacity to “shut it down” when faced with extremely cold temperatures (i.e., based on our measurements of non-mitochondrial O_2_ consumption and Na^+^-K^+^-ATPase–dependent O_2_ consumption; Fig 4C and 4E), and that this ‘hypometabolism’ of cunner hepatocytes may permit vital processes in hepatocytes to occur (e.g., synthesis of antifreeze proteins) without increasing the cell’s overall energy demands.

### Effect of cold temperature on ATP-consuming and producing processes and target mechanisms for metabolic depression

To actively suppress their metabolism in response to decreasing water temperatures, fish would need a carefully orchestrated reduction in the energy expenditure of some cellular processes. The only aspect in which salmon mitochondria appeared to show capacity for cold-induced metabolic suppression were the reductions in State 3 and State 4 mitochondrial respiration (Q_10_ > 3.5). The cunner on the other hand, showed reductions in several cellular processes. State 3 and 4 mitochondrial respiration in this study were actively suppressed (Q_10_ > 3.5), RCR was more than halved (Figs 1 and 2), and O_2_ consumption due to non-mitochondrial activity and Na^+^-K^+^-ATPase fell considerably between 10 and 0°C (Fig 4C and 4E). Further, these changes were in addition to the large reduction in State 4 mitochondrial respiration from 10 to 5°C (Fig 1C), and the previously reported drastic reduction in the protein synthesis [19, 35] of this species at cold temperatures. It is likely that all these changes (and possibly others) are necessary in order to reach the level of metabolic depression needed for cunner to survive the cold winter environment in which they do not feed.

The lower State 4 respiration rates in the cunner vs. the salmon, and at 0 *vs*. 20°C, suggest that the mitochondrial membranes of this species have a lower proton conductance and are less “leaky” in the cold. Fish mitochondrial membranes are among the leakiest of vertebrates, and thus, provide an excellent mechanism to reduce energy expenditure during metabolic depression [45]. However, State 4 respiration is a rudimentary indicator of proton leak and membrane permeability, and thus, we performed parallel measurements of membrane potential and O_2_ consumption (i.e., succinate oxidation) to fully assess if changes in mitochondrial membrane permeability were responsible for the reduction in proton leak [26]. Proton leak rates were successfully measured at 5°C, a first in a study of this kind, as most literature in this area has measured proton leak at temperatures above 20°C (see review by [23]). In both species, liver mitochondria showed a decrease in O_2_ consumption as membrane potential decreased (Fig 3). This indicates an actual reduction in proton conductance, as opposed to a reduction in the reactions of the electron transport chain, as has been shown in frogs and snails [27, 46]. A decrease in mitochondrial membrane permeability has been previously reported in rat hepatocytes [47], but this is in contrast to most other studies which indicate that the reduction in proton leak during hibernation is due to a reduction in substrate oxidation (see review by [23]).

Reductions in enzyme activity and ion pumping offer additional potential mechanisms of saving energy when faced with harsh environmental conditions. Liver CS activity was similar in 0°C and 10°C acclimated cunner (Table 2). However, although Na^+^-K^+^-ATPase activity was ~ 30% (but not significantly) lower at 0°C compared to 10°C in both groups of cunner, it was significantly lower when compared to salmon at 0°C. Finally, O_2_ consumption that is independent of oxidative phosphorylation (e.g., oxidase activity, reactive oxygen species production etc.) which is known to represent up to 70% of O_2_ consumption in trout [25], was significantly reduced (by 40–45%) in cunner tested at 0 *vs*. 10°C whereas it was not different in salmon between assay temperatures (Fig 4C and 4D).

### Effects of thermal acclimation on the metabolic response to decreasing temperature

In both species, the effects of acclimation temperature were limited to temperature-dependent effects on State 3 respiration (Fig 1B), with this parameter having greater temperature sensitivity in 0°C acclimated fish from 10–5°C (Q_10_ values of 9.6 and 3.9, respectively), but a reduced Q_10_ value (of 0.9 *vs*. 5.9) from 5–0°C (Fig 1C). It is difficult to compare these values with the literature as no other data on mitochondrial respiration are available on other salmonids, or ‘active’ temperate fishes, when acclimated or acutely exposed to temperatures approaching 0°C. The only relevant data available is on the *in vivo* MO_2_ of this species measured in 8 and 1°C acclimated fish *vs*. those exposed to an acute 8 to 1°C drop in temperature. These authors [48] did not report any differences in MO_2_ between the two 1°C groups or in the Q_10_ values between acclimated fish and those exposed to the acute temperature decrease. However, such differences may exist in maximum MO_2_ (this parameter the most likely to reflect changes in State 3 mitochondrial respiration), and experiments examining the effects of the above temperatures on cardiorespiratory parameters in salmon during a critical swimming speed test are currently being performed.

Similar differences were also noted for the cunner, however, the magnitude of the difference in Q_10_ values for State 3 respiration between 5 and 0°C was much greater in this species than in the salmon (Fig 1). For example, the Q_10_ value for 0°C acclimated cunner was an incredible 141.2 *vs*. 14.5 in 10°C acclimated fish (Fig 1A and 1B). Indeed, previous studies on cunner suggest that geographic location / latitude (i.e., differences in seasonal thermal ranges) influence the thresholds for the onset of metabolic depression and the degree of metabolic depression in this species. For example, cunner from a more southern population (Woods Hole) had high Q_10_ values (of 5.5–8.9) when water temperature was decreased from 12 to 6.4°C [17], while fish at the most northern limits of this species’ distribution (Newfoundland, [16]) had a Q_10_ value for MO_2_ of 10.4 when water temperature was decreased from 5 to 0°C. Indeed, the particularly large Q_10_ values reported in this study may be necessary for this species to survive in Newfoundland, where it is living at the extreme northern limit of its latitudinal distribution. In Newfoundland, this species is typically exposed to temperatures at < 2°C for approx. 5–6 months of the year, must maintain high antifreeze protein levels to ensure it doesn’t freeze, and doesn’t eat during this period [13, 43, 44, 49].

## Conclusions and perspectives

In this study we show that: 1) cunner mitochondria had much lower values for State 3 and 4 mitochondrial respiration and hepatocyte MO_2_ as compared to Atlantic salmon at all acclimation and assay temperature combinations; 2) mitochondrial State 3 respiration and RCR fall dramatically in the cunner between 10 and 5°C independent of their acclimation temperature; and 3) hepatocyte MO_2_ does not reflect the temperature-dependent changes in mitochondrial respiration, non-mitochondrial MO_2._ or the energy consumed by Na^+^-K^+^-ATPase activity. These are extremely novel data, and provide compelling evidence that metabolic depression occurs in cunner at the mitochondrial and cellular level, and that alterations in mitochondrial State 3 and 4 respiration can be initiated solely by a change in their thermal environment.

Clearly, the results presented here question the conclusion of Speers-Roesch et al. [8] that the reduction in MO_2_ in cunner at cold temperatures (and likely other fish species) is solely related to dormancy (i.e., the suppression of activity), and agree with others [19–21] that metabolic depression is a major component of the large reduction in MO_2_ when some fish are exposed to environmental stressors. There are a number of possible reasons why Speers-Roesch et al. [8] came to a different conclusion based on their findings: that the MO_2_ of cunner acclimated to 0.6°C and acutely exposed to this temperature were not different as compared to fish acclimated to 6.2°C; and that when activity was taken into account Q_10_ values for MO_2_ generally ranged between 2.8 and 3.2. First, the results of our studies clearly show that the cunner’s mitochondria and hepatocytes can downregulate their MO_2_ very quickly; i.e. they are not dependent on the time of exposure to low temperatures. Second, the Q_10_ values reported by Speers-Roesch et al. [8] (2.8–3.2) are on the borderline of being indicative of temperature-dependent effects alone *vs*. of also being influenced by other processes. Third, the *in vivo* MO_2_ of adult cunner as used in Speers-Roesch et al. [8] begins to decrease by 7°C [18], and thus, it is likely that metabolic depression had already been initiated in their fish (i.e., their Q_10_ values were an underestimate). Finally, the liver represents approx. 35–50% of whole animal MO_2_ [26], and the lack of a Q_10_ effect on liver MO_2_ (as suggested by the hepatocyte data in this study, and likely related to antifreeze production [43, 44]), probably concealed / obscured that metabolic depression was occurring in the cunner’s other tissues.

Another key finding of this work was the lack of agreement between the effects of temperature changes on mitochondrial respiration *vs*. hepatocyte MO_2_. This finding appropriately highlights that changes in mitochondrial respiration / function at thermal extremes do not necessarily translate into constraints on cellular or organismal performance, and support the concerns expressed by Chung and Schulte [50] with regard to extrapolating changes in mitochondrial function to the whole animal level. Clearly, cellular control mechanisms can supersede the direct effects of temperature on mitochondrial function. Finally, this research does not investigate, or identify, the mechanisms that were responsible for the rapid and substantial reductions in State 3 respiration in the cunner and in 0°C acclimated salmon from 10–5°C. There are a number of mechanisms, other than those investigated in this study that could be involved (incl. changes in substrate oxidation and transport, sudden changes in membrane fluidity, the regulation of uncoupling proteins, or the formation of mitochondrial permeability transition pores; [50]). Future research should include studies of the potential involvement of these mechanisms, and take a more comprehensive approach to understanding the effects of temperature on fish mitochondrial function / processes, and their influence on whole animal metabolism and performance at temperature extremes. Such information will be critical to understanding how some fish populations will be impacted by climate change, which is expected to be accompanied by more frequent and severe heat waves and cold events [51–53].
